# Antimicrobial stewardship in low- and middle-income countries: Access versus adherence

**DOI:** 10.1371/journal.pmed.1005218

**Published:** 2026-08-28

**Authors:** Kiarah Shchepanik, Anthony D. Bai, Heather Wise

**Affiliations:** 1 Antimicrobial Stewardship Program, Department of Pharmacy Services, Kingston Health Sciences Centre, Kingston, Ontario, Canada; 2 Division of Infectious Diseases, Department of Medicine, Ǫueen’s University, Kingston, Ontario, Canada

## Abstract

This Perspective by Kiarah Shchepanik and colleagues discusses results from the recent LAMPA trial, in which a smartphone app did not improve adherence to antimicrobial prescriber guidelines despite enhanced access, and outline why multifaceted and sustained interventions are needed due to complex prescriber behavior.

## Teaser

Can improving access to antimicrobial guidelines improve prescriber adherence? In a recent study published in *PLOS Medicine* (the LAMPA trial), a smartphone app did not improve adherence despite enhanced access to guidelines, highlighting that prescriber behavior is complex, and effective antimicrobial stewardship interventions need to be multifaceted and sustained.

The World Health Organization (WHO) recognizes antimicrobial resistance (AMR) as a top global public health threat of the 21st century. AMR disproportionately impacts low- and middle-income countries (LMICs); bacterial infections harboring AMR are estimated to cause 5 million deaths annually, of which 4.3 million occur in LMICs [1]. The high infectious disease burden and health system constraints in LMICs create an environment in which inappropriate antimicrobial use and the development of resistance can flourish [1].

The WHO Global Action Plan on AMR [2] identifies antimicrobial stewardship as a critical strategy to preserve the effectiveness of existing antimicrobials. Central to stewardship efforts is the implementation of evidence-based antimicrobial prescribing guidelines that support clinicians in selecting the appropriate antimicrobial, dose, and duration of therapy.

In a recent study published in *PLOS Medicine*, the Lao AntiMicrobial Prescribing Application (LAMPA) trial [3] studied the implementation of a smartphone antibiotic guidelines application in Laos, an LMIC. This stepped-wedge cluster randomized controlled trial was conducted across six hospitals from 2021 to 2022. All hospitals started with a guideline introduction session and a paper-based version of national prescribing guidelines as the reference standard. Then the intervention—a smartphone application of the same guidelines accompanied by one antimicrobial stewardship training session—was implemented sequentially to two hospitals at a time over three-step periods. The primary outcome was the proportion of antimicrobial prescriptions that were adherent to guidelines based on regular point prevalence surveys. The overall guideline adherence was 17.0% (95% CI 15.0%–19.1%) in the reference group and 25.6% (95% CI 23.1%–28.3%) in the intervention group. After accounting for time, cluster effect, and potential confounders, the intervention did not significantly improve guideline adherence, with an adjusted odds ratio of 1.26 (95% CI 0.8–1.9, *p* = 0.276).

This study provides important evidence that smartphone-based dissemination of national antimicrobial prescribing guidelines is feasible across hospitals in an LMIC. The limited resources setting likely influenced how the study was designed and carried out. For example, the repeated point prevalence survey offered a feasible and low-cost alternative to continuous prospective surveillance while still generating longitudinal data that is reproducible by other LMICs [4].

A strength of the study is that the authors chose to assess both inpatient and outpatient antimicrobial prescribing. This broader perspective enhances the applicability of the findings, recognizing that antimicrobial prescribing practices and stewardship challenges differ between inpatient and ambulatory care. However, the timing of the intervention during the peak of the COVID-19 pandemic in Laos represents a limitation, as it delayed the intervention, disrupted implementation and could have affected antibiotic prescribing. Another limitation is that the study did not measure the change in individual prescriber behavior.

The principal finding is that improving access to antimicrobial prescribing guidelines through a smartphone application was insufficient to improve guideline adherence. This reinforces that passive dissemination of clinical guidelines rarely produces improvements in clinician practice without complementary strategies that actively engage prescribers. Effective antimicrobial stewardship requires a multipronged approach that combines guideline availability with ongoing education, clinician engagement, and continuous reinforcement [5,6]. Overall, the study findings should not be interpreted as a failure of digital stewardship tools, but rather as evidence that technology is best used to support, rather than replace, comprehensive stewardship programs.

The low adherence to guidelines despite accessible guidelines in the LAMPA trial [3] is notable with many possible explanations. Physicians rely on clinical experience and established prescribing habits when making antimicrobial decisions, often placing greater trust in these than in evidence-based guidelines [7,8]. Greater clinical experience can increase confidence in prescribing decisions, even when these diverge from guideline recommendations, while knowledge gaps may encourage the continued use of familiar broad-spectrum antibiotics [7,8]. In hospital settings, prescribing decisions may also be influenced by concerns about responsibility for patient outcomes, particularly in surgical practice, where fear of post-operative infections and potential litigation can favor defensive prescribing over guideline-concordant care [7].

In a related study that interviewed 16 prescribers at two Lao hospitals on the new antibiotic guidelines, prescribers were generally satisfied with the simplicity and accessibility of the guidelines [9]. Yet, the prescribers only each accessed the smartphone application approximately once per month in the LAMPA trial [3]. The low level of engagement may reflect perceived barriers to using app-based decision support, including limited familiarity or confidence with the platform, or the fact that many prescribers were unable to attend the training session. Many clinicians perceived little additional value in consulting the guidelines because they relied on established clinical experience or believed they were already familiar with the recommendations [9], which aligns with the broader literature on barriers to guideline adherence, including prescribing habits, clinical experience, organizational culture, and leadership support [10].

In many LMICs, antimicrobial prescribing is also influenced by broader health system constraints. In the LAMPA trial, almost all antibiotic prescriptions were empiric [3]. Limited access to diagnostic microbiology means that many treatment decisions remain empiric [11], reducing opportunities to de-escalate therapy based on culture results. Additionally, shortages of trained healthcare professionals and stewardship resources often preclude the multidisciplinary support that underpins successful antimicrobial stewardship programs in high-income settings [5].

Beyond healthcare infrastructure, challenges related to medicine quality may also influence prescribing practices. An estimated 10% of antimicrobials marketed in LMICs are substandard or falsified**,** with medicines containing incorrect amounts of active ingredient or undergoing degradation because of inadequate transportation and storage conditions [12]. In such environments, clinicians may have reduced confidence in the effectiveness of recommended first-line therapies, creating an additional barrier to guideline-concordant prescribing.

Together, the results from the LAMPA trial demonstrate that technology can help provide better access to antibiotic prescribing guidelines, however, improving access alone does not improve guideline adherence, because factors driving prescriber behavior are complex [3]. Accordingly, effective antimicrobial stewardship interventions need to be multifaceted and sustained. Beyond prescribers, health system challenges in LMICs, such as limited microbiology laboratory support and poor antibiotic quality, also negatively affect antibiotic prescribing and should be addressed. In LMICs, national AMR action plans should consider investments into microbiology lab capacity and regulatory agencies that prevent, detect, and respond to substandard and falsified antibiotics.
